# Symmetries among Multivariate Information Measures Explored Using Möbius Operators

**DOI:** 10.3390/e21010088

**Published:** 2019-01-18

**Authors:** David J. Galas, Nikita A. Sakhanenko

**Affiliations:** Pacific Northwest Research Institute, 720 Broadway, Seattle, WA 98122, USA

**Keywords:** information, entropy, interaction-information, multi-information, Möbius inversion, lattices, multivariable dependence, symmetric group, MaxEnt, networks

## Abstract

Relations between common information measures include the duality relations based on Möbius inversion on lattices, which are the direct consequence of the symmetries of the lattices of the sets of variables (subsets ordered by inclusion). In this paper we use the lattice and functional symmetries to provide a unifying formalism that reveals some new relations and systematizes the symmetries of the information functions. To our knowledge, this is the first systematic examination of the full range of relationships of this class of functions. We define operators on functions on these lattices based on the Möbius inversions that map functions into one another, which we call Möbius operators, and show that they form a simple group isomorphic to the symmetric group S_3_. Relations among the set of functions on the lattice are transparently expressed in terms of the operator algebra, and, when applied to the information measures, can be used to derive a wide range of relationships among diverse information measures. The Möbius operator algebra is then naturally generalized which yields an even wider range of new relationships.

## 1. Introduction

Information-related measures are useful tools for multi-variable data analysis, as measures of dependence among variables, and as descriptions of order and disorder in biological and physical systems. The mathematical relationships among these measures are therefore of significant inherent interest. The description of order and disorder in physical, chemical and biological systems is fundamental. It plays a central role not only in the physics and chemistry of condensed matter, but also in systems with biological levels of complexity, including interactions of genes, macromolecules, cells and of networks of neurons, however it is certainly not well understood. Mathematical descriptions of the underlying order, and transitions between states of order, are still far from satisfactory and a subject of much current research (for example [1,2]). The difficulty arises in several forms, but the dominant contributors are the number and high degree of effective interactions among components, and their non-linearity. There have been many efforts to define information-based measures as a language for describing the order and disorder of systems and the transfer of information. Negative entropy, joint entropies, multi-information and various manifestations of Kullback–Leibler (K–L) divergence are among the key concepts. Interaction information is one of these. It is an entropy-based measure for multiple variables introduced by McGill in 1954 [3] as a generalization of mutual information. It has been used effectively in a number of theoretical developments and applications of information-based analysis [4,5,6,7], and has several interesting properties, including symmetry under permutation of variables. This symmetry is shared with joint entropies and multi-information, though its interpretation as a measure of information in the usual sense is ambiguous as it can have negative values. In previous work we have proposed complexity and dependence measures related to this quantity [8,9]. Here we focus on elucidating the character and source of some of the mathematical properties that relate these measures. The formalism presented here can be viewed as a unification of a wide range of information-related measures in the sense that the relations between them are elucidated. 

This paper is structured as follows: We briefly review a number of definitions and review preliminaries relevant to information measures, lattices and Möbius inversion. In the next section we define operators that map the functions on the lattice into one another, expressing the Möbius inversions as operator equations. We then determine the products of the operators and, completing the set of operators with a lattice complement operator, we show that together they form a group that is isomorphic to the symmetric group, $S_{3}$. In the next section we express previous results in defining dependency and complexity measures in terms of the operator formalism, and illustrate relationships between many commonly used information measures, like interaction information and multi-information. We derive a number of new relations using this formalism, and point out the relationship between multi-information and certain maximum entropy limits. This suggests a wide range of maximum entropy criteria in the relationships inherent in the operator algebra, which are not further explored here. The next section focuses on the relations between these functions and the probability distributions underlying the symmetries. We then illustrate an operator equation expressing our dependence measure in terms of conditional log likelihood functions. Finally, we define a generalized form of the fundamental inversion relation, and show how these operators on functions can be additively decomposed in a variety of ways.

## 2. Preliminaries

We review briefly the elements of information theory and lattices that are relevant to this paper, and clarify some notational conventions used.

### 2.1. Information Theory

Consider a set of n discrete variables $\nu_{n}=\left\{X_{1},X_{2},\ldots ,\;X_{n}\right\}$ denoted as ν if there is no ambiguity. We use $\nu_{n-1}$ to denote the set $\nu_{n}$ without variable $X_{n}$. $\mathrm{Pr}\left(\nu_{n}\right)$ denotes a joint probability density function over $\nu_{n}$, and $\mathrm{Pr}\left(X_{n}|\nu_{n-1}\right)$ denotes a conditional probability density function.

*Marginal entropy* of a single variable $X_{i}$ is defined as $H\left(X_{i}\right)=-\sum_{x_{i}}\mathrm{Pr}\left(x_{i}\right)\log\left(\mathrm{Pr}\left(x_{i}\right)\right)$. Similarly given a set of variables $\nu_{n}$, *joint entropy* is defined as $H\left(\nu_{n}\right)=-\sum_{s}\mathrm{Pr}\left(s\right)\log\left(\mathrm{Pr}\left(s\right)\right)$, where s traverses all possible states of $\nu_{n}$. We write $H\left(X_{n}|\nu_{n-1}\right)$ to denote *conditional entropy* of $X_{n}$ on the rest of the variables $\nu_{n-1}$, obtained by using the conditional distribution and averaging with respect to the marginal. The difference in joint entropy of sets of variables with and without $X_{n}$ is called *differential entropy*$\delta H\left(\nu_{n}\right)$:

(1) $$\delta H\left(\nu_{n}\right)\equiv H\left(\nu_{n}\right)-H\left(\nu_{n-1}\right)$$

The *mutual information*$I\left(X_{i},X_{j}\right)$ measuring the mutual dependence between two variables $X_{i}$ and $X_{j}$ is defined as

(2) $$I\left(X_{i},X_{j}\right)\equiv -\sum_{x_{i},x_{j}}\mathrm{Pr}\left(x_{i},x_{j}\right)\log\left(\frac{\mathrm{Pr}\left(x_{i},x_{j}\right)}{\mathrm{Pr}\left(x_{i}\right)\mathrm{Pr}\left(x_{j}\right)}\right)$$

Equivalently, the mutual information can be expressed *via* marginal and joint entropies:

(3) $$I\left(X_{i},X_{j}\right)\equiv H\left(X_{i}\right)+H\left(X_{j}\right)-H\left(X_{i},X_{j}\right)$$

Similar to Equation (3), given three variables $X_{i}$, $X_{j}$, and $X_{k}$, the conditional mutual information can be defined as

(4) $$I\left(X_{i},X_{j}|X_{k}\right)=H\left(X_{i}|X_{k}\right)+H\left(X_{j}|X_{k}\right)-H\left(X_{i},X_{j}|X_{k}\right)$$

A generalization of mutual information to more than two variables is called *interaction information* [3]. For three variables it is defined as the difference between mutual information with and without knowledge of the third variable:

(5) $$I\left(X_{i},X_{j},X_{k}\right)=I\left(X_{i},X_{j}\right)-I\left(X_{i},X_{j}|X_{k}\right)$$

When expressed entirely in terms of entropies we have

(6) $$\begin{array}{cc} I\left(X_{i},X_{j},X_{k}\right)= & H\left(X_{i}\right)+H\left(X_{j}\right)+H\left(X_{k}\right)\\ & -H\left(X_{i},X_{j}\right)-H\left(X_{i},X_{k}\right)-H\left(X_{j},X_{k}\right)\\ & +H\left(X_{i},X_{j},X_{k}\right) \end{array}$$

Consider the interaction information for a set of n variables $\nu_{n}$

(7) $$I\left(\nu_{n}\right)=-\sum_{\tau \subseteq \nu_{n}}{\left(-1\right)}^{\left|\tau \right|}H\left(\tau \right)$$

The interaction information $I\left(\nu_{n}\right)$ for a set of n variables obeys a recursion relation that parallels that for the joint entropy of sets of variables, $H\left(\nu_{n}\right)$, which is derived in turn directly from the probability chain rule:

(8) $$\begin{array}{l} H\left(\nu_{n}\right)=H\left(\nu_{n-1}\right)+H\left(X_{n}|\nu_{n-1}\right)\\ I\left(\nu_{n}\right)=I\left(\nu_{n-1}\right)-I\left(\nu_{n-1}|X_{n}\right) \end{array}$$

where the second terms on the right are conditionals. These two information functions are known to be related by Möbius inversion [4,5,6,7].

Given Equation (7), we define the *differential interaction information*, Δ, as the difference between values of successive interaction informations arising from adding variables

(9) $$\Delta \left(\nu_{n-1};X_{n}\right)\equiv I\left(\nu_{n}\right)-I\left(\nu_{n-1}\right)=-I\left(\nu_{n-1}|X_{n}\right)$$

The last equality in Equation (8) comes from the recursive relation for the interaction information, Equation (5). The differential interaction information is based on providing the target variable $X_{n}$ to be added to the set of n−1 variables, and is therefore asymmetric. If we multiply differential interaction informations with all possible choices of the target variable, the resulting measure is symmetric and we call it a *symmetric delta*, $\bar{\Delta }$

(10) $$\bar{\Delta }\left(\nu_{n}\right)={\left(-1\right)}^{n}\prod_{X\in \nu_{n}}\Delta \left(\nu_{n}-\left\{X\right\};X\right)$$

There is another measure for multivariable dependence called *multi-information*, or *total correlation* [10], which is defined as the difference between the sum of single entropies for each variable of a set and the joint entropy for the entire set

(11) $$\Omega \left(\nu_{n}\right)\equiv \sum_{X_{i}}H\left(X_{i}\right)-H\left(\nu_{n}\right)$$

Multi-information is frequently used because it is always postive and goes to zero when all the variables are independent. We can think of it as a kind of conglomerate of dependencies among members of the set $\nu_{n}$. At the two-variable level multi-information, Kullback–Leibler divergence and interaction information are all identical, and equal to mutual information. There is an inherent duality between the marginal entropy functions and the interaction information functions based on Möbius inversion, which we will show in detail in Section 3. Bell described an elegantly symmetric form of the inversion and identified the source of this duality in the lattice associated with the variables [4]. The duality is based on the inclusion lattice of the set of variables. We start with this symmetric inversion relation and extend it to an algebra of operators on these lattices. We will first define the lattice and other relevant concepts from lattice theory before discussing Möbius inversion further. 

### 2.2. Lattice Theory

We review here some definitions from the lattice theory that we will use [11]. We say that a set P is a *poset* (a *partially ordered set*) if there is a partial order defined on it, 〈P,≤〉. A partial order (≤) is a binary relation that is reflexive, antisymmetric, and transitive. Note that we would write x≤y to denote the partial order between elements x and y of a poset. Note also that an inverse of a partial order is a partial order. A *chain* of a poset 〈P,≤〉 is a subset C⊆P such that for any two elements x,y∈C either x≤y or y≤x. Similarly, a path of length k is a subset C⊆P such that $C=\left[x_{1},x_{2},\ldots ,x_{k}\right]$ for any 1≤i<k either $x_{i}\leq x_{i+1}$ or $x_{i+1}\leq x_{i}$. Note that any chain is a path, but not other way around, since $x_{i}$ and $x_{j}$ of a path need not be ordered if |i−j|>1.

Let X be a subset of a poset 〈P,≤〉. The *minimum* of X, if exists, is min(X) such that min(X)∈X and for any x∈X:min(X)≤x. Similarly, the *maximum* of X, if exists, is max(X) such that max(X)∈X and for any x∈X:x≤max(X). A poset 〈P,≤〉 has a *top* element (a greatest element) T iff T∈P and for any x∈P:x≤T. Similarly, a poset 〈P,≤〉 has a *bottom* element (a least element) ⊥ iff ⊥∈P and for any x∈P:⊥≤x.

The *dual* of a poset 〈P,≤〉 is 〈P,≥〉, where ≥ is the inverse partial order of ≤. For any statement based on the partial order ≤ and true about all posets, the dual statement (based on the inverse partial order ≥) is also true about all posets.

For a poset 〈P,≤〉 we call D⊆P a *down-set* (or an *ideal*) iff, for any d∈D:∀p∈P:(p≤d)⇒(p∈D). Dually, we call U⊆P an *up-set* (or a *filter*) iff for any u∈U:∀p∈P:(p≥u)⇒(p∈U). Note that a set S is a down-set of 〈P,≤〉 iff its set complement P\S is an up-set of 〈P,≤〉.

Given a subset S of a poset P,≤, M is an *upper bound* of S iff for any x∈S:x≤M. And dually, m is a *lower bound* of S iff for any x∈S:m≤x. The *join* of S, if exists, is called an upper bound of S, which is the least of the upper bounds of S. And dually, the *meet* of S is the greatest lower bound of S.

A poset where for every two elements there exist the unique join and meet is called a *lattice*. A lattice that contains a top element T and a bottom element ⊥, such that for every element x of the lattice, ⊥≤x≤T, is called a *bounded lattice*. An *inclusion lattice* (also called a *subset lattice*) is a typical example of a lattice defined on all subsets of a given set S ordered by a subset inclusion ⊆. If a set S is finite, then its corresponding inclusion lattice is bounded, where the top element is S itself and the bottom element is the empty set. 

## 3. Möbius Dualities 

Many applications make use of the relations among information theoretic quantities like joint entropies and interaction information that are formed by what can be called Möbius duality [4]. Restricting ourselves to functions on subset lattices, we note that a function on a lattice is a mapping of each of the lattice element (subset of variables) to the reals. The Möbius function for this lattice is $\mu \left(\nu ,\tau \right)={\left(-1\right)}^{\left|\nu \right|-\left|\tau \right|}$ where τ is a subset of ν, |τ| is the cardinality of the subset.

### 3.1. Möbius Inversion 

Consider a set of n variables ν and define g, the dual of f for the set of variables:

(12a) $$g\left(\eta \right)=\sum_{\tau \subseteq \eta }\mu \left(\nu ,\tau \right)f\left(\tau \right)=\sum_{\tau \subseteq \eta }{\left(-1\right)}^{\left|\nu \right|-\left|\tau \right|}f\left(\tau \right);\;\eta \subseteq \nu$$

Note that function g is the interaction information if f were the entropy function H, adopting the sign convention of [4]. It can easily be shown that the symmetric relation holds:

(12b) $$f\left(\eta \right)=\sum_{\tau \subseteq \eta }{\left(-1\right)}^{\left|\nu \right|-\left|\tau \right|}g\left(\tau \right);\;\eta \subseteq \nu$$

The relations defined in Equation (12a,b) represent a symmetric form of Möbius inversion, and the functions f and *g* can be called Möbius duals, and inversions of one another.

Now consider an inclusion lattice. The Möbius inversion is a convolution of the Möbius function with any function defined on the lattice over all its elements (subsets) between the argument subset, τ, of the function and the empty set. The summation in the inversion is over all the elements on all chains between τ and the empty set, counting the elements only once, which is called a down-set of the inclusion lattice (see Section 2). The empty set, at the limit of the range of the convolution, can be considered as the “reference element”. We use the idea of a reference element in Section 7 in generalizing the inversion relations. The range of the convolution can of course be limited at the top element (largest subset) and the bottom element of the lattice. In defining the Möbius operators below we need to carefully define how the range is determined.

**Figure 1:**
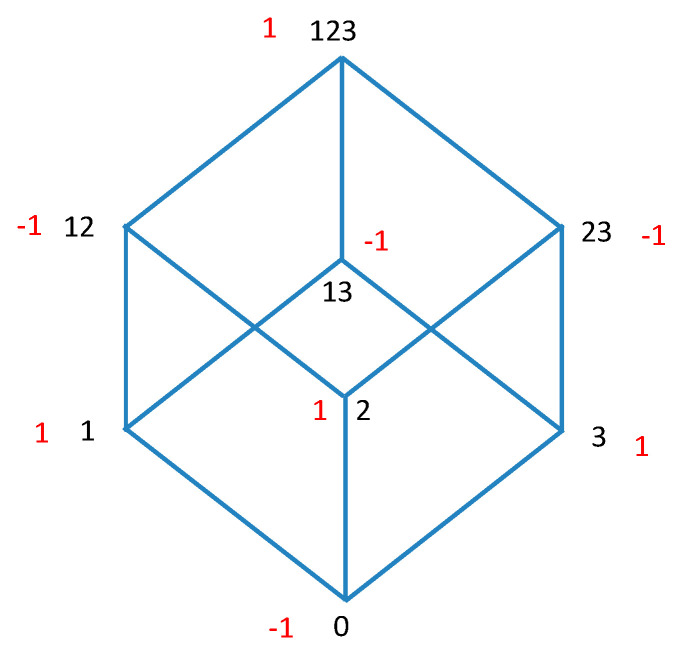
The Hasse diagram of the subset lattice for three variables. The numbers in black are the variable subsets, while the Möbius function μ(ν,τ) on this lattice (1 or −1) is indicated in red.

To illustrate the relations concretely the nodes and the Möbius function are shown graphically for three variables in Figure 1. When the functions in Equation (12) are mapped onto the lattice for three variables, these equations represent the convolution of the lattice functions and the Möbius function over the lattice.

### 3.2. Möbius Operators

The convolutions with the Möbius function over the lattice in Equation (12) define mappings that can be expressed as operators. The operators can be thought of as mapping of one function on the lattice into another. A function on the lattice, in turn, is a map of the subsets of variables at each node into the real numbers. When acting on sums or differences of functions the operators are distributive.

**Definition** **1.** Möbius down-set operator. *Given a set of variables,*τ*, which is the element in the inclusion lattice, we define the Möbius* down-set operator, $\hat{m}$, *that operates on a function on this lattice.*

(13a) $$\hat{m}\left(f\left(\tau \right)\right)\equiv \sum_{\eta \subseteq \tau }{\left(-1\right)}^{\left|\eta \right|-1}f\left(\eta \right)=g\left(\tau \right),\;\tau \subseteq \nu$$

*The down-set operator is defined as an operator form of the convolution with the Möbius function: the sum over the lattice of* subsets of τ*, of product of the values of the function times the Möbius function. The upper bound of this convolution is the entire set,*τ*, the lower bound is the empty set.*

Likewise, we can define a Mobius *up-set operator*. The definition is significantly different in that the lower limit needs to be specified, whereas the downset operator uses the empty set unless otherwise specified.

**Definition** **2.** Mobius up-set operator. *Given a set of variables,*ν*, the operator,*$\hat{M}$*, is defined as the convolution operator on a function on the inclusion lattice which is the sum is over the lattice of supersets of*τ.

(13b) $$\hat{M}\left(f\left(\tau \right)\right)\equiv \sum_{\eta ⊇\tau }{\left(-1\right)}^{\left|\eta \right|+1}f\left(\eta \right)=h\left(\tau \right),\;\eta ,\tau \subseteq \nu$$

*The lower bound of this convolution is*τ *and the upper bound is the complete set*ν.

Given a function, f, Equations (13a,b) define the functions g and h, respectively: the *down-set* and *up-set* inverses, or duals, of f. The sum in the expression of Equation (13a) is the same as the symmetric form of the Möbius inversion [4]: f and g in Equation (13a) are interchangable, dual with respect to the down set operator (see Equations (12a) and (12b)). Given a subset argument of the function, the up-set operator induces a convolution whose limits are the given subset and the full set, while the down-set operator’s convolution’s limits are the given subset and the empty set. 

**Figure 2:**
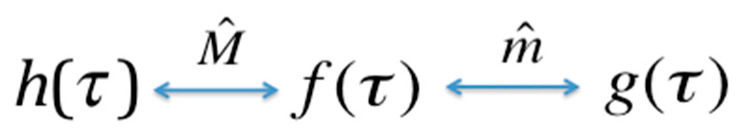
The Möbius operators define the duality relationships between the functions on the subset lattice.

We see from Equation (13a) that the nature of Möbius inversion implies that the down-set operator applied twice yields the identity, ${\hat{m}}^{2}=\hat{I}$. Similarly, using Equation (13b) we see that ${\hat{M}}^{2}=\hat{I}$. This is an expression of the duality: this idempotent property of the Möbius operators is equivalent to the symmetry in Equation (12); in other words, the exchangability in these equations, or duality of the functions is exactly the same property as the idempotecy of the operators. The relationships between pairs of the dual functions, generated by the operators are shown in the diagram in Figure 2. The range of the convolution operators is clear here, but this will not always be true, and where it is ambiguous we use a subscript on the operator to identify the reference set. We will need this subscript in Section 7.

To advance this formalism further we need to define another operator on the inclusion lattice. The complementation operator, $\hat{X}$, has the effect of mapping function values of all elements of the lattice (subsets) into the function values of the corresponding set complement elements. For example, node 1 maps into node 23 in Figure 1, as 23 is the complement of 1 for the three element set. Viewed in 3D as a geometric object, as shown in Figure 1, the complementation corresponds to an inversion of the lattice, all such 3-D coordinates mapping into their opposites through the origin at the geometic center of the cube. We thus define the operator $\hat{X}$, acting on functions whose arguments are subsets τ of the set ν:

(14) $$\hat{X}f\left(\tau \right)={\left(-1\right)}^{\left|\nu \right|}f\left(\tilde{\tau }\right):\;\tau \subseteq \nu ,\;\tau \cap \tilde{\tau }=\emptyset ,\;\tau \cup \tilde{\tau }=\nu$$

The sign change factor is added since inversion of the lattice also has the effect of shifting the Möbius function by a sign for odd numbers of total variables on the lattice. 

**Figure 3:**
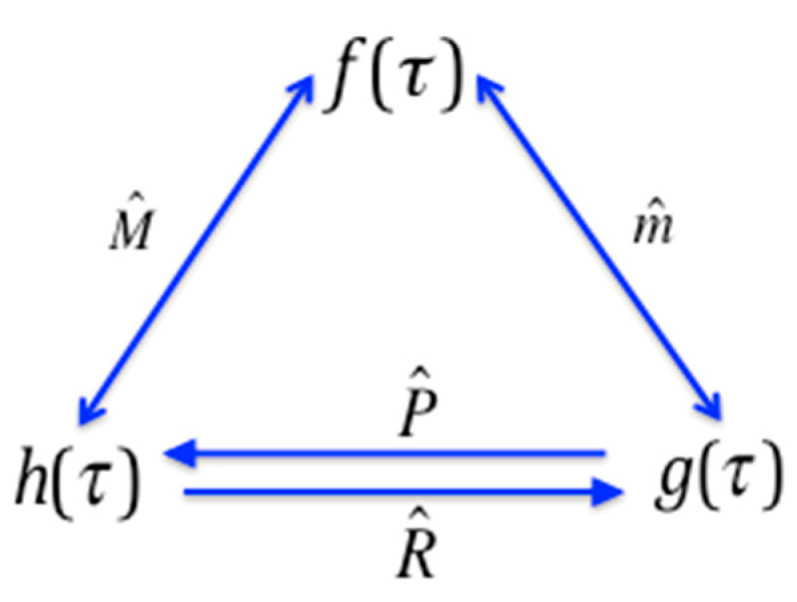
Diagram of the mappings of the functions on the subset lattice into one another by the operators. The operators $\hat{P}$ and $\hat{R}$ are: $\hat{P}=\hat{X}\hat{M},\;\hat{R}=\hat{X}\hat{m}$.

If we define the composite operators, $\hat{P}$ and $\hat{R}$, as:

(15) $$\hat{P}=\hat{X}\hat{M},\;\hat{R}=\hat{X}\hat{m}$$

the pairwise relations among the functions and the operators shown in Figure 3 then follow. The three- and four-variable case for the relationships in Figure 3 can easily be confirmed by direct calculation, and as it happens the general case is also easy to prove. The proofs are direct and follow from the Möbius inversion sums, by keeping track of the effects of each of the inversion and convolution operators, and are not presented here.

Let us now collect the operators of Figure 3, add the identity operator and the composite operators $\hat{P}$ and $\hat{R}$, and calculate the full product table of the set of operators. This product table of the operators is shown in Table 1.

It is immediately clear that this set of six operators forms a group: the set is closed, it contains an identity element, all its elements have an inverse included, and they demonstrate associativity. Furthermore, examination of the table immediately shows that it is isomorphic to the symmetric group $S_{3}$, the group of permutations of three objects.

Table 2 shows the 3 × 3 matrix representation of the group $S_{3}$, with the one-line notation of the operator effect, and the correspondence between the Möbius operators and the $S_{3}$ representation. 

Note that while the operators themselves, which act on functions, depend on the number of variables since they define convolutions, their relationships do not. Thus, the group structure is independent of the number of variables in the lattice. For any number of variables the structure is simply the permutation group, $S_{3}$.

## 4. Connections to the Deltas

The differential interaction information and the symmetric deltas were defined in [8] as overall measures of both dependence and complexity (see definitions in Equations (9) and (10)). We will now show the connection between these deltas and our operator algebra. We will use the three-variable case to illustrate the connection. First recall that if the marginal entropies are identified with the function f, then by the definitions of the down-set operator (Equation (13a)) and the interaction information (Equation (7)) we have:

(16a) $$\hat{m}\left(H\left(\nu \right)\right)\equiv \sum_{\eta \subseteq \nu }{\left(-1\right)}^{\left|\eta \right|-1}H\left(\eta \right)=I\left(\nu \right)$$

which for three variables using simplified notation is:

(16b) $$\hat{m}\left(H_{123}\right)\equiv H_{1}+H_{2}+H_{3}-H_{12}-H_{13}-H_{23}+H_{123}=I_{123}$$

If the marginal entropies are identified with the function f in Equation (12), and the interaction informations identified with g, then the differential interaction information is identified with h. For the three-variable case these examples are shown using simplifed notation:

(16c) h(1)=Δ(23;1), h(2)=Δ(13;2), h(3)=Δ(12;3)

Simplifying the notation we can express the relations between these functions using the Möbius operator as:

(16d) $$\Delta \left(\tau ;X\right)=\hat{M}H\left(X\right)=-I\left(\tau |X\right)$$

The full set of the lattice is τ∪{X}  and the variable X is singled out as in Equation (16c). Furthermore, the convolution can be seen to take place over the set τ∪{X}. Equation (16d), if interpreted properly, provides a simple connection between the deltas and the Möbius operator algebra, and expresses a key relation (Theorem 1). We have proved the following theorem.

**Theorem** **1.** *The Möbius* up-set *operator acting on the* join-irreducible elements *of the lattice of marginal entropies generates the conditional interaction informations, the deltas, for the full set of variables of the lattice.*

*Join-irreducible* lattice elements are all those that cannot be expressed as the join, or union, of other elements. In this case they are all the single variables. Since the deltas are differentials of the interaction information at the top of the lattice (the argument of the function is the full set), their expression in terms of the join-irreducible elements is the most fundamental form. 

**Figure 4:**
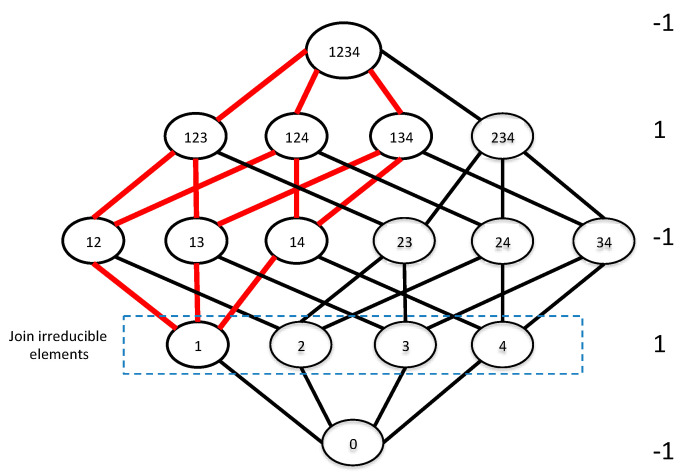
The four-variable lattice showing the 4 join-irreducible elements that generate the symmetric deltas as in Equation (16c). Möbius function values are shown on the right, and the red lines connect the elements of the delta function, Δ(234;1), which form a 3D-cube.

To illustrate the relation more concretely, Figure 4 shows the specific connection between the join-irreducible elements and deltas for the four-variable lattice. A general statement of this connection emerging from this geometric picture is a general property of the algebraic structure of the subset lattice.

**Corollary** **1.** *The differential of one function on the lattice corresponds to the up-set operator on another function of the join-irreducible elements.*

Written in terms of the functions related by the inversions, and using the same set notation as above, X indicating a join-irreducible element, we can state this general result as follows.

If $g\left(\tau \right)=\hat{m}f\left(\tau \right)$ and X is a join-irreducible element of lattice, then:

(17) $$\hat{M}f\left(X\right)=h\left(\tau ;X\right)=g\left(\tau |X\right)$$

where the final term is a conditional form of the g function in which X is instantiated. This is defined as function over all τ for which X∈τ. These deltas, and delta-like functions more generally, are represented as convolutions over a lattice that is one dimension less than the full variable set lattice. 

We have previously proposed the symmetric delta (the product of all variable permutations of the delta function, h) as a measure of complexity, and of collective variable dependence [8]. Then the symmetric delta, simply the product of the individual deltas, is seen to be the product of the results of the up-set operator acting on the functions of *all of the join-irreducible elements of the entropy lattice*. Note that by Equation (8) both the conditional entropies and conditional interaction informations, since they correspond to the differentials, imply a path independent chain rule. Note that these kinds of differential functions include many more than just those generated by the up-set operator acting on the join-irreducible elements, as shown in the next section. 

## 5. Symmetries Reveal a Wide Range of New Relations

The system of functions and operators defined in the previous section reveals a wide range of relationships. Examination of Equation (8) and comparision with 16d shows that delta is also related to the differential entropy (defined by Equation (1)) measuring the change in the entropy of a set when we consider an additional variable. Keep in mind that the differential is defined by the full set and the added variable. Applying the down-set operator to Equation (1), and using sets $\nu_{n}$ and $\nu_{n-1}$ as the upper bounds, gives us:

**Theorem** **2.** *Given the definition of the differential entropy (the difference in joint entropy of sets of variables with and without*$X_{n}$, $\delta H\left(\nu_{n}\right)\equiv H\left(\nu_{n}\right)-H\left(\nu_{n-1}\right)$*), and the definitions of the up-set and down-set operators, and their distributive character over functions on the lattice:*

(18) $$\begin{array}{l} \hat{m}\delta H\left(\nu_{n}\right)=\hat{m}\left(H\left(\nu_{n}\right)-H\left(\nu_{n-1}\right)\right)=I\left(\nu_{n}\right)-I\left(\nu_{n-1}\right)=-I\left(\nu_{n}|X_{n}\right)\\ \hat{m}\delta H\left(\nu_{n}\right)=\hat{M}\left(H\left(X_{n}\right)\right) \end{array}$$

*where*$X_{n}$*is the element that is the difference between the sets $\nu_{n}$ and $\nu_{n-1}$. *

Equation (18) is based on the successive application of the differential and the down-set operators (recall from Equations (16a) and (16b) that $\hat{m}\left(H\left(\nu \right)\right)=I\left(\nu \right)$). Each of these acts on and produces a function of a subset of variables on the lattice, so their effects are well defined.

We can consider δ as an operator, if we define the additional variable that is added to obtain $\nu_{n}$, but note that it does not define a convolution over elements of the lattice as do the Möbius operators. Considering δ as an operator (recalling that it is defined by two sets differening by a single variable) we note that δ and $\hat{m}$ commute. The duality between H and I implies a dual version of Equation (18) as well, which we will not derive here. If we apply other operators to the expression in Equaton (18) we find another set of relations among these marginal entropy functions. For example, another remarkable symmetry emerges:

(19) $$\begin{array}{l} \delta H\left(\nu_{n}\right)=\hat{m}\hat{M}H\left(X_{n}\right)=\hat{X}\hat{m}H\left(X_{n}\right)=\hat{R}H\left(X_{n}\right)\\ H\left(X_{n}\right)=\hat{P}\delta H\left(\nu_{n}\right) \end{array}$$

This can easily be checked for three and four variables by direct calculation, and by referring to the group Table 1. Equations (18) and (19) are seen to relate functions of the higher lattice elements to functions of the join irreducible elements. 

There are further symmetries to be seen in this set of information functions. Consider the mapping diagram of Figure 3. If we define a function which is simply the delta function with each lattice element mapped into its set complement, that is, acted on by the lattice complementation operator, from Equation (16d) we have (supressing the argument notation in the functions):

(20) $$\Phi \equiv \hat{X}\Delta ,\;\hat{X}\hat{m}\Phi =\hat{X}\hat{m}\hat{X}\Delta =H$$

**Figure 5:**
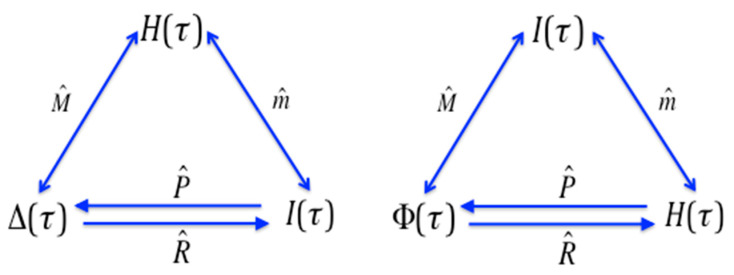
A simple modifcation of one of the functions by lattice complementation modifies the postion of functions in the mapping diagram. The original diagram is on the left, the result of Δ modified by complementation is on the right.

Then these functions occupy different positions in the mapping diagram as seen in Figure 5. Several other such modifications can be generated by similar operations. 

There are a large number of similar relations that can be generated by such considerations. There are other information-based measures that we can describe using the operator algebra. Because it is a widely used measure for multi-variable dependence we will now examine the example of multi-information, Ω, defined by Equation (11). In terms of entropy functions on the lattice elements, Ω, as expressed in this equation, can be thought of as the sum of the join-irreducible elements, minus the top element or the join of the inclusion lattice. To apply the down-set operator to the terms in Equation (11) we must carefully define the bounds of the convolutions. If we calculate the convolution over the Ω function, we have:

(21) $$\hat{m}\Omega \left(\nu_{n}\right)=\sum_{X_{i}}\hat{m}H\left(X_{i}\right)-I\left(\nu_{n}\right)$$

Since the upper bound of the down-set operator is defined as the argument set of the function, the down-set of a single variable function is the function itself (since $H(X_{i})-0=H(X_{i})$). Note that we are using the distributive property of the operator here. The application of the up-set operator to the multi-information function on the lattice, on the other hand, gives us:

(22) $$\hat{M}\Omega \left(\nu_{n}\right)=\sum_{X_{i}}\Delta \left(\nu_{n-1};X_{i}\right)-H\left(\nu_{n}\right)$$

Since the multi-information is a composite function the results of the action of the (distributive) Möbius operators are also composite functions.

## 6. Relation to Probability Densities

### 6.1. Conditional log Likelihoods and Deltas

Writing the differential entropy in terms of the probability distributions, using the definitions of the joint entropies and the probability chain rule, gives:

(23a) $$\delta H\left(\nu_{n}\right)=-\langle \ln\frac{\mathrm{Pr}\left(\nu_{n}\right)}{\mathrm{Pr}\left(\nu_{n-1}\right)}\rangle =-\langle \ln\mathrm{Pr}\left(X_{n}|\nu_{n-1}\right)\rangle =H\left(X_{n}|\nu_{n-1}\right)$$

For simplicity of notation we define π as the expectation value on the right. We have:

(23b) $$\pi \left(X_{n}|\nu_{n-1}\right)\equiv -\langle \ln\frac{\mathrm{Pr}\left(\nu_{n}\right)}{\mathrm{Pr}\left(\nu_{n-1}\right)}\rangle =\langle \ln\mathrm{Pr}\left(\nu_{n-1}\right)-\ln\mathrm{Pr}\left(\nu_{n}\right)\rangle$$

From Equation (23) we see that π is a conditional log likelihood function. By applying the down-set operator, $\hat{m}$, to π we generate some interesting relations. As seen in Equations (18), the result of this operation is the delta, the conditional interaction information,

(24) $$\hat{m}\pi \left(X_{n}|\nu_{n-1}\right)=\hat{m}\delta H\left(\nu_{n}\right)=\hat{M}H\left(X_{n}\right)=-I\left(\nu_{n-1}|X_{n}\right)=\Delta \left(\nu_{n-1};X_{n}\right)$$

Expressing this in another way, using the group table, we have the expressions from Equation (19), and therefore:

(25a) $$\pi \left(X_{n}|\nu_{n-1}\right)=-\langle \ln\mathrm{Pr}\left(X_{n}|\nu_{n-1}\right)\rangle =\delta H\left(\nu_{n}\right)=\hat{R}H\left(X_{n}\right)$$

The expected value of the log of the probability of a given, single variable, conditioned on the other variables in the subset, can therefore be expressed simply in terms of Möbius operators acting on the entropy functions of a lattice. This is the principal result of this section, embodied in Theorem 3.

**Theorem** **3.** *The symmetric delta is the product of all conditional log likelihood functions acted on by the down-set operator:*

(25b) $$\bar{\Delta }\left(\nu_{n}\right)=\prod_{\mathrm{all}\;\mathrm{choices}\;\mathrm{of}\;X_{n}}\Delta \left(\nu_{n-1};X_{n}\right)=\prod_{\mathrm{all}\;\mathrm{choices}\;\mathrm{of}\;X_{n}}\hat{m}\pi \left(X_{n}|\nu_{n-1}\right)$$

The relation of the π’s to the deltas is clear here, and the subsets of the variables under consideration can then generate a series of conditional log likelihoods (CLL’s) for $\left|\nu_{m}\right|=m$, $\left\{\pi \left(X_{m}|\nu_{m-1}\right)\right\}$ for m≥2. The simplest approximation for dependencies among variables is realized in the case m=2, where CLL’s are approximated by those with a single conditional variable. In this case (using simplified notation):

(26a) $$\begin{array}{c} \pi \left(2|1\right)=H_{12}-H_{1}\\ \pi \left(3|1\right)=H_{13}-H_{1} \end{array}$$

and we have for the three-variable case:

(26b) $$\begin{array}{l} \Delta \left(23;1\right)=H_{1}-H_{12}-H_{13}+H_{123}=-\pi \left(2|1\right)+\pi \left(2|13\right)\\ \Delta \left(23;1\right)=-\pi \left(3|1\right)+\pi \left(3|12\right) \end{array}$$

There are two different ways to express deltas as sums of the π’s. Several conclusions follow from these considerations. Since the group table for the Möbius operators exhibits several different, equivalent operators, $\hat{R}=\hat{m}\hat{M}=\hat{X}\hat{m}=\hat{M}\hat{X}={\hat{P}}^{2}$, we can express the correspondence between Δ and the CLL’s in several equivalent ways. These expressions then provide direct links with other information functions.

### 6.2. Towards Prediction

An approach to extracting relations predictive of a variable from the information in a data set is suggested by the above considerations. The general problem can be defined as how to determine the “best” prediction formula for the value of one variable in the set, say $X_{1}$, from analysis of a data set of all variables. We sketch the suggested approach here. Step one of the process, is to define the problem by determining the maximum degree of dependence to be considered, that is to determine the number of variables involved. Step two is to calculate the symmetric deltas to determine which sets of variables are dependent on one another [9]. Step three is to find the maximum expected CLL, from the set $\left\{\pi \left(X_{1}|X_{i}\right),\pi \left(X_{1}|X_{i},X_{j}\right),\pi \left(X_{1}|X_{i},X_{j},X_{k}\right)\ldots \right\}$ by calculating the expectations of the entropy differentials. Note that the specific, expected entropy differences tend to zero as the dependence of the single variable, $X_{1}$, on the other variables increases. Finally, once the “best” likelihood function is found, a predictive function is estimated based on the data: an estimate of the probabilities of $X_{1}$ conditioned on all the other variables of the set. The general framework for inference is clear. This procedure is reminiscent of the Chow-Liu algorithm [12] which is entirely pairwise and based on mutual information. Our approach provides a direct way towards generating predictive rules from large, multivariable data sets. We will develop this approach further in a future paper. 

## 7. Generalizing the Möbius Operators

The up-set and down-set operators, $\hat{M}$ and $\hat{m}$, defined above, generate convolutions over chains from each element of the inclusion lattice to the top element (full set) or to the bottom element (empty set) respectively. The convolutions are either “down”, towards subset elements, or “up” toward supersets. The chains over which the convolutions (sums of the product of function and Möbius function) are taken are clear and are defined by the subset lattice for these two operators. No element is included more than once in the sum. Moreover, the sign of the Möbius function is the same across all elements at the same distance from the extreme elements.

We can generalize the Möbius operators by defining the range of the convolution, the end elements of the paths, to be any pair of elements of the lattice, an upper and lower element, rather than one of them being defined by the bounds of the lattice. Two elements are required: the starting element, and an ending element. The starting element is determined by the argument of the function being operated on, but the ending element can be defined to generalize the operators. We can call the ending element a *reference element*. The specification of both the upper and lower element is essential here. For example, instead of the up-set operator, with the full set ν as its natural reference element, we could designate an arbitrary subset element like {1,2} as the reference and thereby define another operator. Consider now a lattice of the full set ν, where η designates a reference element.

**Definition** **3.** *The generalized Möbius operator $F_{\eta }$, acting on a function of a subset, f(τ), τ⊆ν, is defined by Equation (27), where the subsets of variables, ς, ranges over all of the shortest paths between τ and η. The functions f(ς) only occur once in the sum, of course, even if they are on more than one path, as for the original operators:*

(27) $$F_{\eta }f\left(\tau \right)=\sum_{\begin{array}{cc} \varsigma \; & \mathrm{on}\;\mathrm{all}\;\mathrm{shortest}\;\mathrm{paths}\\ & \mathrm{between}\;\tau \;\mathrm{and}\;\eta \end{array}}{\left(-1\right)}^{\left|\nu \right|-\left|\varsigma \right|}f\left(\varsigma \right)$$

*There are often multiple shortest paths between any two elements in the lattice, since the subset lattice is a hypercube. We specify the upper and lower elements by the reference and the element specified by the function. *

The two extreme reference elements, the empty set and the full set, then yield the down-set and up-set operators respectively:

(28) $$\begin{array}{c} F_{0}f=\hat{m}f\\ F_{\nu }f=\hat{M}f \end{array}$$

The reference element η establishes a relation between the lattice sums and the Möbius function. It is the juxtaposition of the lattice, anchored at η, to the Möbius function that defines the symmetries of the generalized Möbius operator algebra. Note that we now have the possibility of including elements that are not ordered along the paths by inclusion since the reference element can be chosen from any lattice element. For example, the convolution between {1} and {2,3} for the 3D-cube lattice, shows this clearly (see Figure 1) as it inclues {1,2}, {2} and the empty set.

**Definition** **4.** *Given μ,η⊆ν we define the complement generalized Möbius operator as ${\tilde{F}}_{\mu }\equiv \hat{X}F_{\mu }\hat{X}$.*

The products of the generalized operators can easy be calculated for the 3- and 4-element sets. We can identify some similarities of these general operators to the operators $\hat{M}$ and $\hat{m}$. First, we note that the operators, $F_{\mu }$, are all idempotent. This is easy to calculate for the 3D and 4D case, and to derive using the relations indicated in Equation (27). The idempotent property implies that there are pairs of functions that are related by each general Möbius operator – a generalized Möbius inversion on the inclusion lattice, a generalized duality. Furthermore, the products exhibit other familiar symmetries. The notable relationships that involve a subset and its complement are summarized in the following theorem.

**Theorem** **4.** *For all μ,η⊆ν the following properties of the generalized Möbius operator and its complement hold: *

(29a) $$F_{\mu }F_{\eta }={\tilde{F}}_{\eta }{\tilde{F}}_{\mu }$$

(29b) $$F_{\mu }{\tilde{F}}_{\eta }=F_{\eta }{\tilde{F}}_{\mu }$$

(29c) $$F_{\mu }=-{\tilde{F}}_{\tilde{\mu }}$$

(29d) $$F_{\mu }f\left(\tilde{\mu }\right)=F_{\eta }f\left(\tilde{\eta }\right)$$

*where $\tilde{\mu }$ and $\tilde{\eta }$ are set complements of μ and η correspondingly.*

Equation (29a) is true since the products of the generalized Möbius operators involve the operator $\hat{X}$, namely $F_{\mu }F_{\eta }=\hat{X}F_{\eta }F_{\mu }\hat{X}$, which in the geometric metaphor is like a rotation of the hypercube (inclusion lattice). Applying Equation (29a) to $F_{\mu }{\tilde{F}}_{\eta }$ results in Equation (29b). The property shown in Equation (29c) follows directly from the definition of $F_{\mu }$ and its complement. The proof of the last property (Equation (29d)) is direct as follows. Since the limiting elements of the convolution are a subset and its complement, it encompasses the whole lattice. Thus $F_{\gamma }f\left(\tilde{\gamma }\right)$ for any subset γ is seen to describe the convolution over all subsets of the entire lattice and therfore Equation (29d) holds. 

**Figure 6:**
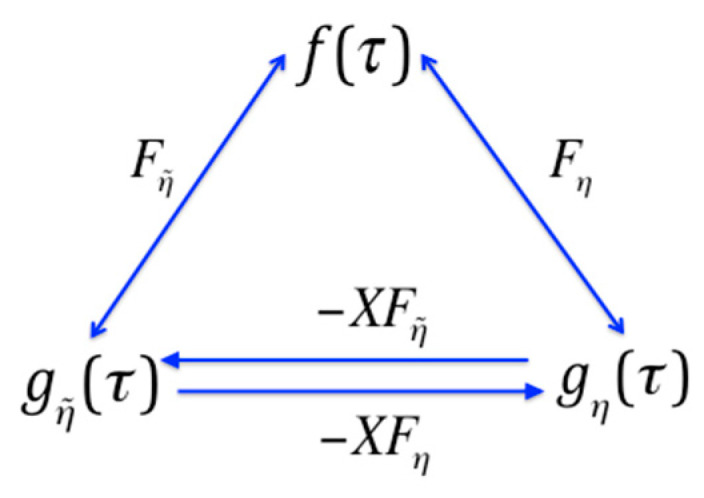
Generalized Möbius operator relations. A diagram of the relations among the functions as determined by the operators. The upper two arrows represent the generalized Möbius inversion relations. The function $g_{\eta }\left(\tau \right)$ is the designation of the function created by the operator $F_{\eta }$. The $S_{3}$ structure is reflected in the similarity with the diagram of Figure 3. Note that when η=∅ the figure becomes identical to Figure 3.

The full group structure of the general operator algebra is more complex than the group defined by the up-set and down-set operators as there are many more operators, defined by the full range of reference elements. (If N is the number of subsets on the lattice there are N−1 down-set operators, while for the generalized case there are ${\left(N-1\right)}^{2}$ operators). The symmetry of the subgroups determined by pairs of complementary subsets are preserved, remaining isomorphic to $S_{3}$ (seen to be true for the 3D, and 4D case by direct calculation, and it appears to be generally true, though we do not yet have a proof of the general case). The relations between these pairs of functions on the lattice is described by the diagram in Figure 6. It appears that the sets of three functions, specific to a reference set η, with the operators that map one into the other exhibit the same overall symmetries reflected in the group $S_{3}$. The pairs of operators identified with a subset and its complement are the key elements of the group. This is because this particular combination of operator and function defines a convolution over the entire set, ν. This identity therefore includes the specific up-set and down set relations, and is equal to the interaction information if f is the entropy function. 

**Figure 7:**
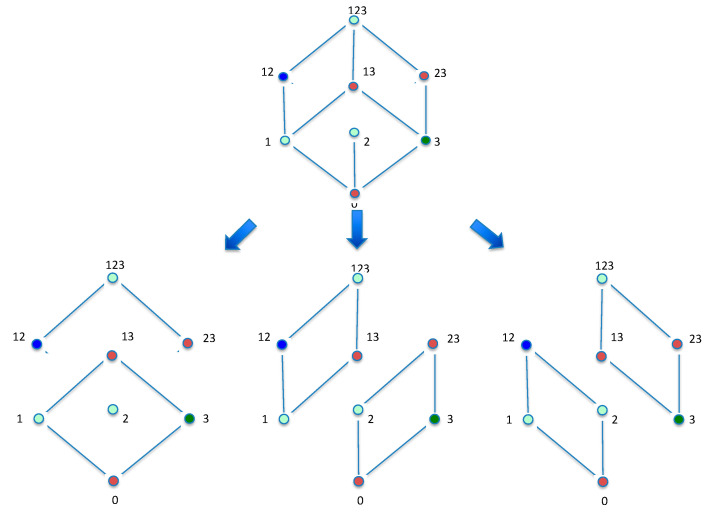
Decomposing the 3D-cube Hasse diagram into two squares (2D hypercubes), by passing a plane through the center of the cube three different ways.

We now ask if sums of such operator-function pairs can be used to decompose a convolution. This decomposition issue can be addressed by asking this specific question: are there sums of operators acting on functions that add up to a given specific operator acting on another function? If this is possible how do we decompose such convolutions and what do they mean? The simple decomposition of the hypercube into sub-lattices can be shown to be equivalent to the process of finding these convolutions, or operator decompositions. We will not deal with the decomposition relations in a general dimension here, but rather demonstrate them for {1,2,3} and {1,2,3,4}. First, let’s consider the 3D case. There are three possible ways to decompose the 3D-cube Hasse diagram into two squares (2D hypercubes), which is done by passing a plane through the cube parallel to the faces (see Figure 7).

Considering one of these decompositions (the leftmost decomposition in Figure 7) results in the following:

(30) $$F_{0}f_{123}=F_{2}f_{123}+F_{0}f_{13}$$

Each of the two terms on the right-hand side could be expressed in operator terms in eight ways (each of the four elements of the sub-lattice being a reference element). There are thus a total of 192 decompositions of the full 3-set convolution, 64 per each of the three decompositions of the cube into two squares. Note that each decomposition leads to the same set of functions, but it is a distinct operator expression. For the 4-set decomposition, there are four ways of decomposing the 4-hypercube into two cubes, so the total number of possible decompositions is 4 × 192 × 192 = 147,456.

## 8. Discussion

Many diverse information measures have been used in descriptions of order and dependence in complex systems and as data analysis tools [3,4,5,6,7,8,9,13,14]. While the mathematical properties and relationships among these information-related measures are of significant interest in several fields, there has been, to our knowledge, no systematic examination of the full range of relationships and symmetries, and no unification of this diverse range of functions into a single formalism as we do here. Beginning with the known duality relationships, based on Möbius inversions of functions on lattices, we define a set of operators on functions on subset inclusion lattices that map the functions into one another. We show here that they form a simple group, isomorphic to the symmetric group $S_{3}$. A wide range of relationships among the set of functions on the lattice can be expressed simply in terms of this operator algebra formalism. When applied to the information-related measures they can express many relationships among various measures, providing a unified picture and allowing new ways to calculate one from the other using the subset lattice functions. For example, we can express the conditional mutual information in the 4D, {1,2,3,4} lattice as sums of convolutions of entropy functions with a few terms for multiple 3D and 2D lattices, or create new information functions with specific symmetries and desired properties. Much is left to explore in the full range of implications of this system, including algorithms for prediction from complex data sets, and other ways in which these functions may be used or computed.

This formalism allows us also to make connections with other areas where lattices are useful. Since any distributive lattice is isomorphic to the lattice of sets ordered by inclusion, all the results presented here apply to any system of functions defined on a distributive lattice [11,15]. Therefore this unification extends well beyond the information measure functions. Distributive lattices are widespread and include the following: every Boolean algebra is a distributive lattice; the Lindebaum algebra of most logics that support conjunction and disjunction is a distributive lattice; every Heyting algebra is a distributive lattice, every totally ordered set is a distributive lattice with *max* as the join and *min* as the meet. The natural numbers also form a distributive lattice with the greatest common divisor as the meet and the least common multiple as the join (this infinite lattice, however, requires some extension of the equivalence proof).

The relationships shown here unify, clarify, and can serve to guide the use of a range of measures in the development of the theoretical characterization of information and complexity, and in the algorithms and estimation methods needed for the computational analysis of multi-variable data. Recently Bettencourt and colleagues have used the conditional form of the interaction information (Equation (8)) to generate an expansion which they used to identify subgraphs in complex networks [16]. This expansion can be viewed as the series of successive delta functions obtained by increasing number of variables and the size of the lattice. The concept of using an expanding lattice (adding variables) that enables such expansions is a very interesting connection to our formalism that will be explored in future work.

We have addressed the relationships between the interaction information, the deltas (conditional interaction information), and the underlying probability densities. We find that the deltas can be expressed as Möbius sums of conditional entropies, the multi-information is simply related by the operators to other information functions, and we made an initial connection to the maximum entropy method. We also note that Knuth has proposed generalizations of the zeta and Möbius functions that define *degrees of inclusion* on the lattices [17,18]. Knuth’s formalism, integrated with ours, could lead to a more general set of relations, and add another dimension to this theory by incorporating uncertainty or variance in the information-related measures. This could be particularly useful in developing future methods for complexity descriptions and data analysis. 

Since the information-related functions have been directly linked to interpretations in algebraic topology [19] it will also be interesting to explore in future work the topological interpretation of the Möbius operators.

From the simple symmetries of these functions and operators it is clear there is more to uncover in this complex of relationships. The information theory-based measures have a surprising richness and internal relatedness in addition to their practical value in data analysis. While we have described here a systematic structure of relationships and symmetries, the full range of possible relationships, insights and applications using Möbius pairs of functions remains to be fully explored. The practical value of this complex of relationships of information measures will be further evaluated using specific examples in our future publications.

## Figures and Tables

**Table 1:** The product table for the six operators above. The operators $\hat{P}$ and $\hat{R}$ are defined as $\hat{P}=\hat{X}\hat{M},\;\hat{R}=\hat{X}\hat{m}$. The convention is that the top row is on the right and the left column on the left in the products indicated; e.g., $\hat{M}\hat{X}=\hat{R},\hat{X}\hat{M}=\hat{P}$. The orange indicates the identity operator.

		Right					
		$\hat{I}$	$\hat{m}$	$\hat{X}$	$\hat{M}$	$\hat{P}$	$\hat{R}$
**Left**	$\hat{I}$	$\hat{I}$	$\hat{m}$	$\hat{X}$	$\hat{M}$	$\hat{P}$	$\hat{R}$
	$\hat{m}$	$\hat{m}$	$\hat{I}$	$\hat{P}$	$\hat{R}$	$\hat{X}$	$\hat{M}$
	$\hat{X}$	$\hat{X}$	$\hat{R}$	$\hat{I}$	$\hat{P}$	$\hat{M}$	$\hat{m}$
	$\hat{M}$	$\hat{M}$	$\hat{P}$	$\hat{R}$	$\hat{I}$	$\hat{m}$	$\hat{X}$
	$\hat{P}$	$\hat{P}$	$\hat{M}$	$\hat{m}$	$\hat{X}$	$\hat{R}$	$\hat{I}$
	$\hat{R}$	$\hat{R}$	$\hat{X}$	$\hat{M}$	$\hat{m}$	$\hat{I}$	$\hat{P}$

**Table 2:** The 3 × 3 matrix representation of symmetric group $S_{3}$ and the corresponding Möbius operators. The one-line notation on the left shows the permutations.

One-line Notation:(Permutation)	Matrix Representation (Left Action Convention)	Möbius Operator
123	$\left(\begin{array}{ccc} 1 & 0 & 0\\ 0 & 1 & 0\\ 0 & 0 & 1 \end{array}\right)$	$\hat{I}$
213	$\left(\begin{array}{ccc} 0 & 1 & 0\\ 1 & 0 & 0\\ 0 & 0 & 1 \end{array}\right)$	$\hat{m}$
132	$\left(\begin{array}{ccc} 1 & 0 & 0\\ 0 & 0 & 1\\ 0 & 1 & 0 \end{array}\right)$	$\hat{M}$
321	$\left(\begin{array}{ccc} 0 & 0 & 1\\ 0 & 1 & 0\\ 1 & 0 & 0 \end{array}\right)$	$\hat{X}$
231	$\left(\begin{array}{ccc} 0 & 1 & 0\\ 0 & 0 & 1\\ 1 & 0 & 0 \end{array}\right)$	$\hat{P}$
312	$\left(\begin{array}{ccc} 0 & 0 & 1\\ 1 & 0 & 0\\ 0 & 1 & 0 \end{array}\right)$	$\hat{R}$
